# Reported Changes in Dietary Behavior Following a First Clinical Diagnosis of Central Nervous System Demyelination

**DOI:** 10.3389/fneur.2018.00161

**Published:** 2018-03-20

**Authors:** Rebecca D. Russell, Robyn M. Lucas, Vanessa Brennan, Jill L. Sherriff, Andrea Begley, The Ausimmune Investigator Group, Caron Chapman, Lucinda J. Black

**Affiliations:** ^1^School of Public Health, Curtin University, Perth, WA, Australia; ^2^National Centre for Epidemiology and Population Health, Research School of Population Health, The Australian National University, Canberra, ACT, Australia; ^3^Centre for Ophthalmology and Visual Science, University of Western Australia, Perth, WA, Australia

**Keywords:** ausimmune study, diet, multiple sclerosis, nutrition, dietary behavior

## Abstract

**Background/objectives:**

Although the current evidence is insufficient to recommend a special diet for people with multiple sclerosis (MS), dietary advice for people with MS is prolific online and in the media. This study aimed to describe dietary changes made in the year following a first clinical diagnosis of central nervous system demyelination (FCD), a common precursor to MS.

**Subjects/methods:**

We used follow-up data from the Ausimmune Study, a multicentre matched case-control study examining the environmental risk factors for a FCD. A total of 244 cases (60 male, 184 female) completed a 1-year follow-up interview, which included a question about dietary changes. We described the number and proportion (%) of participants who reported making dietary changes and the type of change made. We investigated independent predictors of making a dietary change using a multivariable logistic regression model.

**Results:**

A total of 38% (*n* = 92) of participants at the 1-year follow-up reported making at least one dietary change over the last year. There were no statistically significant independent associations between any participant characteristic and odds of making a dietary change. Of those who made at least one dietary change, the most common changes were increasing fruit and/or vegetable intake (27%, *n* = 25) and following a low-fat diet (25%, *n* = 23).

**Conclusion:**

A considerable proportion of the study population reported making at least one dietary change in the year following a FCD, with the majority of changes being toward a healthier diet. Further research is warranted to investigate the reasons behind any dietary changes adopted by people with a FCD or with MS, and whether making a dietary change has benefits for the progression of demyelinating diseases, e.g., to a diagnosis of MS, as well as for general health and well-being.

## Introduction

Multiple sclerosis (MS) is a chronic, immune-mediated disease of the central nervous system (CNS), characterized by inflammatory damage of the myelin sheaths that insulate and protect the CNS axons (1). With the exclusion of traumatic brain injury, MS is the most common cause of neurological disability in young adults (2). It is estimated to affect more than 2 million people globally—approximately three quarters of whom are women—and it is currently incurable (3). Although the specific etiology of MS is unknown, both genetic and environmental factors, including higher latitude, low vitamin D status, smoking, and history of infectious mononucleosis, are associated with increased risk of f MS (4).

A number of restrictive diets are promoted online and in the media for people with MS, including the Swank Diet (5) (saturated fat restricted to <20 g/day; unsaturated fat restricted to 20–40 g/day); the Overcoming MS (OMS) Recovery Program (6) (low in saturated fat; moderate in seafood, avocado and nuts; no meat, dairy, egg yolks, or refined foods); and the Paleo diet (high in meat, vegetables, and fruits; no dairy, legumes, or grains) (7). There is little empirical evidence to support the benefits of such dietary modifications for disease activity or progression in MS. However, following a healthy diet (e.g., increasing consumption of vegetables, fruits, whole grains, legumes, nuts and seeds, and oily fish; and limiting red meat, animal products, and highly processed foods) may improve overall well-being in people with MS (8, 9) and reduce the symptoms and co-morbidities associated with MS, such as fatigue and obesity (10).

Using data from the 2003–2006 Australian Multicentre Study of Environment and Immune Function (Ausimmune) Study, we investigated the types of dietary changes made by participants after a first clinical diagnosis of CNS demyelination (FCD), a common precursor to MS. We investigated whether demographic factors or lifestyle characteristics influenced whether a participant made a dietary change or not.

## Materials and Methods

### Study Design and Population

The Ausimmune Study was a multicentre case–control study in four regions of Australia along the south-eastern and eastern seaboard, namely Brisbane city, Newcastle region, Geelong and the Western Districts of Victoria, and Tasmania (11). The study aimed to explore the environmental risk factors for developing a FCD. Further details of the Ausimmune Study have been documented elsewhere (11).

In brief, cases were aged between 18 and 59 years, and diagnosed with a FCD during the study period of November 2003 to December 2006. Study neurologists reviewed all clinical data (including magnetic resonance imaging scans) to confirm the diagnosis and eligibility to participate.

At baseline there were 282 case participants who had a FCD within the study period. This included 216 participants with a classic first demyelinating event within the study period, 18 participants presenting for the first time with primary progressive MS, and 48 participants who had received a first diagnosis of CNS demyelination, but provided a history consistent with a prior neurological event consistent with demyelination, and were thus now diagnosed as having MS.

At 1-year from initial entry into the Ausimmune Study, case participants were invited to complete a telephone interview with a research nurse. A total of 244 cases completed the 1-year telephone review, providing data for this analysis. Of those, 186 had had a classic FDE at baseline, 44 had had a prior neurological event, and 14 had been diagnosed with primary progressive MS. Written informed consent was obtained from all participants and they were free to withdraw from the study at any time. Nine regional Human Research Ethics Committees approved the Ausimmune Study.

### Data Collection

#### Baseline Data

Participants completed questionnaires prior to, and during, a face-to-face interview with a research nurse. Data were collected on participant characteristics, including age, sex, ethnicity, education, usual physical activity in the last 12 months when the person was not ill [using the International Physical Activity Questionnaire (IPAQ)] (12) and known risk factors for MS, such as smoking and history of infectious mononucleosis. At the interview, a study nurse measured height and weight, and a blood sample was taken by venepuncture.

#### 1-Year Follow-Up

At the 1-year review, participants were asked: “Have you changed your diet over the last year? If yes, how?” This was an open field question where the responses were coded by the interviewer into seven categories as per the coding guide: more fish/high fish, more vitamin D, low-fat diet, low-dairy diet, high-fiber diet, special MS diet (e.g., Swank diet), and other. The “other” category was a free text field. Participants could describe more than one dietary change.

### Statistical Analysis

Ethnicity was categorized as Caucasian or other. Physical activity was categorized as low, medium, or high physical activity, according to the IPAQ scoring protocol (12). Body mass index (BMI) was calculated as weight in kilograms divided by height in metres squared and categorized as underweight, normal, overweight, and obese, using the standard cut-points according to ethnicity (13). Only two participants were categorized as underweight; hence, underweight participants were combined with the normal weight group for analysis purposes. Dietary changes that were reported by at least five participants (including those captured in the free text field) were used to create ten final dietary change categories: (1) increased fruit and/or vegetables; (2) low-fat diet; (3) reduced discretionary foods, including alcohol; (4) increased fish; (5) reduced meat; (6) reduced or eliminated gluten/wheat; (7) high fiber diet; (8) low dairy diet; (9) increased water/fluids; and (10) reduced saturated fat intake. Dietary changes that were reported by fewer than five participants were grouped into “other.”

Using the number and proportion (%), we described the characteristics (sex, age at follow up, ethnicity, study region, education at baseline, physical activity at baseline, BMI category at baseline, smoking at baseline) of those who did and did not make a dietary change. Missing data for characteristics were as follows: ethnicity, *n* = 2; education, *n* = 2; physical activity, *n* = 19; BMI category, *n* = 1; and smoking, *n* = 3. We described the number and proportion (%) of participants making specific types of dietary changes. We used univariate logistic regression models to investigate potential predictors of making a dietary change. In addition, we used a multivariable logistic regression model (*n* = 221) to investigate potential independent predictors of making a dietary change, with all variables included in a single model. Data were analyzed using SPSS statistical software for Windows (Version 23.0, IBM Corp., Armonk, NY, USA) and Stata Statistical Software (Release 14, College Station, TX, USA: StataCorp LP). Statistical significance was defined as *P* < 0.05.

## Results

The characteristics of the 244 participants with 1-year follow-up data are shown in Table 1. Over one-third of participants (*n* = 92, 38%), reported making at least one dietary change in the year following a FCD. There were no statistically significant associations between any participant characteristic and the odds of making a dietary change in the univariate logistic regression models (Table 2), nor in the multivariable logistic regression model (Table 3). Obesity at baseline was associated with a 60% increase in the odds of making a dietary change compared to being normal weight/underweight, although the association did not reach statistical significance (Table 3).

**Table 1:** Characteristics of participants (*n* = 244) in the 1-year follow-up of the Ausimmune Study according to whether or not they made at least one dietary change in the year after a first clinical diagnosis of CNS demyelination.

	Made at least one dietary change (*n* = 92)	Made no dietary changes (*n* = 152)
**Sex, % (*n*)**		
Male	22.8 (21)	25.7 (39)
Female	77.2 (71)	74.3 (113)
**Age group at follow-up, % (*n*)**		
20–29 years	17.4 (16)	19.1 (29)
30–39 years	39.1 (36)	29.0 (44)
40–49 years	30.4 (28)	34.9 (53)
50–59 years	13.0 (12)	17.1 (26)
**Ethnicity, % (*n*)**		
Caucasian	97.8 (89)	96.0 (145)
Other	2.2 (2)	4.0 (6)
**Study region, % (*n*)**		
Brisbane (27°S)	29.4 (27)	25.7 (39)
Newcastle (33°S)	17.4 (16)	12.5 (19)
Geelong (37°S)	28.3 (26)	25.0 (38)
Tasmania (43°S)	25.0 (23)	36.8 (56)
**Education at baseline, % (*n*)**		
Year 10 or less	21.7 (20)	28.7 (43)
Year 12 and TAFE	54.4 (50)	47.3 (71)
University	23.9 (22)	24.0 (36)
**Physical activity at baseline, % (*n*)**		
Low	18.8 (16)	17.1 (24)
Moderate	38.8 (33)	34.3 (48)
High	42.4 (36)	48.6 (68)
**BMI category at baseline, % (*n*)**		
Normal/underweight (≤24.9)	45.7 (42)	43.7 (66)
Overweight (25–29.9)	20.7 (19)	33.8 (51)
Obese class 1 (30–34.9)	18.5 (17)	15.2 (23)
Obese class 2 (≥35)	15.2 (14)	7.3 (11)
**Smoking at baseline, % (*n*)**		
Current smoker	27.5 (25)	30.0 (45)
Past smoker	38.5 (35)	30.0 (45)
Never smoked	34.1 (31)	40.0 (60)

*TAFE, Technical and Further Education*.

*Missing data as follows: ethnicity, *n* = 2; education, *n* = 2; physical activity, *n* = 19; Body mass index (BMI) category, *n* = 1; and smoking, *n* = 3*.

**Table 2:** Univariate logistic regression models^a^ showing associations between each participant characteristic and odds of making a dietary change.

	*n*	OR (95% CI)	*P*
Sex (female vs. male)	244	1.17 (0.64, 2.14)	0.619
Age group at follow-up, years	244		
20–29		Reference	
30–39		1.48 (0.70, 3.15)	0.305
40–49		0.96 (0.45, 2.05)	0.911
50–59		0.84 (0.33, 2.09)	0.703
Ethnicity (other vs. Caucasian)	242	0.54 (0.11, 2.75)	0.461
Study region	244		
Brisbane (27°S)		Reference	
Newcastle (33°S)		1.22 (0.53, 2.78)	0.642
Geelong (37°S)		0.99 (0.49, 1.99)	0.974
Tasmania (43°S)		0.59 (0.30, 1.18)	0.138
Education at baseline	242		
Year 10 or less		Reference	
Year 12 and TAFE		1.51 (0.80, 2.88)	0.205
University		1.31 (0.62, 2.78)	0.476
Physical activity at baseline	225		
Low		Reference	
Moderate		1.03 (0.48, 2.23)	0.938
High		0.79 (0.37, 1.68)	0.547
Body mass index category at baseline	243		
Normal/underweight (≤24.9)		Reference	
Overweight (25–29.9)		0.59 (0.30, 1.13)	0.108
Obese class 1 (30–34.9)		1.16 (0.56, 2.43)	0.690
Obese class 2 (≥35)		2.00 (0.83, 4.82)	0.122
Smoking at baseline	241		
Current smoker		Reference	
Past smoker		1.51 (0.81, 2.80)	0.195
Never smoked		1.08 (0.56, 2.07)	0.828

*TAFE, Technical And Further Education*.

*^a^A separate model was run for each characteristic*.

**Table 3:** A multivariable logistic regression model^a^ showing independent associations between participant characteristics and odds of making a dietary change (*n* = *221*).

	OR (95% CI)	*P*
Sex (female vs. male)	1.23 (0.60, 2.52)	0.575
**Age at follow-up, years**		
20–29	Reference	
30–39	1.43 (0.61, 3.36)	0.417
40–49	0.81 (0.35, 1.89)	0.630
50–59	0.61 (0.21, 1.75)	0.356
Ethnicity (other vs. Caucasian)	1.03 (0.17, 6.12)	0.971
**Study region**		
Brisbane (27°S)	Reference	
Newcastle (33°S)	1.84 (0.71, 4.77)	0.210
Geelong (37°S)	1.16 (0.52, 2.61)	0.711
Tasmania (43°S)	0.92 (0.42, 2.00)	0.824
**Education at baseline**		
Year 10 or less	Reference	
Year 12 and TAFE	1.63 (0.75, 3.55)	0.215
University	1.37 (0.56, 3.33)	0.493
**Physical activity at baseline**		
Low	Reference	
Moderate	1.04 (0.45, 2.41)	0.924
High	0.93 (0.41, 2.15)	0.870
**Body mass index category at baseline**						
Normal/underweight (≤24.9)	Reference	
Overweight (25–29.9)	0.56 (0.26, 1.18)	0.128
Obese class 1 (30–34.9)	1.61 (0.69, 3.73)	0.270
Obese class 2 (≥35)	1.62 (0.56, 4.70)	0.378
**Smoking at baseline**		
Current smoker	Reference	
Past smoker	1.78 (0.86, 3.68)	0.118
Never smoked	1.28 (0.60, 2.76)	0.522

*TAFE, Technical and Further Education*.

*^a^All characteristics included in a single model*.

Table 4 shows the types of dietary changes made by participants. The most common dietary changes were increasing fruit and/or vegetable intake (*n* = 25) and following a low-fat diet (*n* = 23). The descriptions of dietary changes adopted by fewer than five participants are detailed in Table S1 in Supplementary Material. Many of the changes were reported by only one participant, and not all changes were healthy (e.g., increased consumption of discretionary foods).

**Table 4:** Types of dietary changes reported by those participants who made at least one dietary change (*n* = 92).

	Males (*n* = 21)		Females (*n* = 71)	
	%	*n*	%	*n*
Increased fruit and/or vegetables	23.8	5	28.2	20
Low-fat diet	28.6	6	23.9	17
Reduced discretionary foods	33.3	7	16.9	12
Increased fish	9.5	2	16.3	15
Reduced meat	23.8	5	16.9	12
Reduced gluten	19.0	4	12.7	9
High-fiber diet	19.0	4	9.9	7
Low-dairy diet	0	0	11.3	8
Increased fluids	23.8	5	4.2	3
Reduced saturated fat	4.8	1	7.0	5
Other^a^	71.4	15	84.5	60

*Participants could select more than one type of dietary change*.

*^a^See Table S1 in Supplementary Material for a list of other dietary changes made by fewer than five participants*.

## Discussion

A considerable proportion of the study population (38%) reported making at least one dietary change in the year following a FCD. The majority of changes were toward a healthier diet, including a move toward higher vegetable and fruit consumption, following a low-fat diet, and reduced consumption of discretionary foods. This is in contrast to a study of dietary change in the general Australian population, where there was a change in diet over 15 years to a higher energy density diet, particularly among older people (14).

Many types of dietary changes in this study were adopted by fewer than five participants, and most of these were adopted by only one or two people. This indicates a lack of consistency in the dietary changes that people are making after a FCD. It is likely that there is confusion about what changes are beneficial for people with MS and those at high risk of MS, with no consistent advice being provided by clinicians and other health-care professionals. Improvements in the strength of evidence about suitable dietary changes for people with MS or those at high risk of MS, coupled with improved nutrition education, would perhaps result in more consistent dietary changes. Not all changes were toward a healthier diet, with a few participants reporting increased convenience and discretionary food consumption. Greater prevalence of anxiety, depression and fatigue is well-described in people with CNS demyelination and MS (15, 16), and may account for this increase in unhealthy food choices.

To our knowledge, no other studies have investigated dietary changes in people with a FCD; however, some studies have reported dietary changes made by people with MS. In Germany, Schwarz and colleagues reported complementary and alternative medicine (CAM) use, including “diet modification,” in members from the AMSEL (Aktion Multiple Sklerose Erkrankter Landesverband) Baden-Wuerttemberg regional chapter of the German MS Society, based on postal surveys (*n* = 1,573, average disease duration of 14 years) (17). Dietary modification was the most popular type of CAM, with 31% currently following a modified diet (17). Also in Germany, Riemann-Lorenz and colleagues conducted a web-based survey of 337 people with MS who visited the German MS Society website, and found that 42% of participants had previously—or were currently—following a diet since their MS diagnosis (18). Of those who reported dietary changes, 25% modified their fat intake, 21% eliminated meat, 22% followed a vegetarian pattern, and 17% reduced meat and increased fish intake (18). Approximately a quarter of participants reported ending their diet, with reasons such as the diet was too restrictive, too expensive, too much effort, or they felt no effect (18). A smaller study in the United States reported that 17% of participants with MS were currently attempting a diet, and the majority (91%) were willing to attempt dietary modification as a means of benefiting their disease (19). Participants were most willing to follow the Paleo diet or a high-carbohydrate diet (19).

There has only been one Australian study investigating the dietary changes made after a diagnosis of MS. A postal survey was conducted by Leong and colleagues to examine dietary interventions used by people with MS who were recipients of the South Australian MS Society Newsletter (*n* = 416) (20). The number of dietary interventions reported by participants ranged from none to 11, with the median reported as one. A total of 40% of participants reported following a low-fat diet, 24% reduced or eliminated sugar, 16% excluded wheat or gluten, and 11% followed the Swank diet. It is not clear if the dietary modifications reported were concerning current use, or inclusive of lifetime use prior to a diagnosis of MS.

In our study, very few participants reported following a special diet for MS (e.g., the Swank Diet, the OMS Recovery Program and the Paleo diet). These diets may be more commonly adopted by people with diagnosed MS (rather than with a FCD). For example, a study in the United States (*n* = 3,140) showed that 16% of people with MS followed the Swank Diet for an average of 4 years, with the intention to treat overall MS symptoms, fatigue, and loss of appetite (21). The baseline and initial follow-up phases of the Ausimmune Study were completed more than 10 years ago. It is possible that diets such as the OMS Recovery Program and the Paleo diet were less widely publicized at that time, and this may explain the low uptake of these diets. However, the Swank diet has been well-known for many years, yet only one participant reported changing to this diet. Our understanding of diet and MS has progressed little over the past decade; it thus seems likely that the findings of this study remain valid today.

Restrictive diets, particularly those which eliminate entire food groups or those that contain strict limitations on dietary components such as red meat or saturated fats, have the potential to result in vitamin and mineral deficiencies (22, 23). With restrictive diets, health consequences—including exacerbation of fatigue (24)—may arise if there is no appropriate dietary compensation (25). MS Australia does not support a special dietary protocol for MS; rather, it recommends a diet that is high in vegetables, fruits, legumes, and whole grains; moderate in dairy intake; low in saturated fat; and includes oily fish for long chain omega-3 fatty acids (26). This is in line with the Australian Dietary Guidelines (27). Higher consumption of fruits and vegetables may reduce the risk of coronary heart disease and stroke, and may protect against weight gain and some cancers (27). Weight gain is common in people with MS (24) and adopting a healthier diet, such as increasing fruit and vegetable consumption, may help to prevent caloric excess and associated weight gain, which may exacerbate symptoms of MS (28).

Given that fewer than 4% of 19- to 50-year olds in Australia adhere to the Australian Dietary Guidelines (29), improved nutrition education and dietary counseling to help people with MS follow these guidelines may help to enhance their well-being and sense of control, particularly in the early stages of the disease. A needs analysis was conducted in Australian in 2012 and showed that dietetic counseling was a high priority for people with MS; however, many felt that their access to such services was not satisfactory (30). The need for improved access to dietary resources was highlighted—in particular, dietetic services with a focus on MS (30).

A strength of the Ausimmune Study was the multicentre design, which enabled data to be captured along a relatively wide latitudinal span in four regions along the Australian eastern seaboard. Participant recruitment was after a FCD and thus early in the disease course of CNS demyelination, which meant that early dietary changes were captured.

There are several limitations to this study. The categories of dietary change were coded by the interviewing nurse, which may have introduced error through misclassification, especially considering the complexity of the diets being described by participants. Furthermore, self-reported dietary intake carries the inherent risk of social desirability bias, where participants generally overestimate healthy behaviors, and portray a more desirable diet than they actually consume (31). The relatively small sample size may have affected the statistical power of our analyses, reducing our capacity to detect any true predictors of making a dietary change.

In this Australian population, over one-third of participants reported a dietary change in the year following a FCD, largely toward a healthier diet. Demographic or lifestyle factors did not have a statistically significant influence on the reported dietary changes. In order to develop appropriate dietary resources and create targeted nutrition education, it is important to understand why some people with a FCD or with MS change their diet and why others do not, why people choose specific diets, and where they obtain their nutrition information. Future research is warranted to investigate the reasons behind any dietary changes adopted by people with a FCD or MS, and the barriers faced in achieving a healthy diet.

## Ethics Statement

Written informed consent was obtained from all participants and they were free to withdraw from the study at any time. Nine regional Human Research Ethics Committees (HREC) approved the Ausimmune Study.

## The Ausimmune Investigator Group Includes the Following Investigators

**Dr. Caron Chapman**, Barwon Health, Geelong, Victoria, Australia; **Prof. Alan Coulthard**, Royal Brisbane and Women’s Hospital and the University of Queensland, Brisbane, Queensland, Australia; **Prof. Keith Dear**, School of Public Health, University of Adelaide, South Australia, Australia; **Prof. Terry Dwyer**, Murdoch Childrens Research Institute, University of Melbourne, Melbourne, Victoria, Australia; **Prof. Trevor Kilpatrick**, Centre for Neuroscience, University of Melbourne, Melbourne, Australia; **Prof. Robyn Lucas**, National Centre for Epidemiology and Population Health, The Australian National University, Canberra, Australian Capital Territory, Australia; **Prof. Tony McMichael (dec)**, National Centre for Epidemiology and Population Health, The Australian National University, Canberra, Australian Capital Territory, Australia; **Prof. Michael P. Pender**, Royal Brisbane and Women’s Hospital and the University of Queensland, Brisbane, Queensland, Australia; **Prof. Anne-Louise Ponsonby**, Murdoch Childrens Research Institute, University of Melbourne, Melbourne, Victoria, Australia; **Prof. Bruce Taylor**, Menzies Research Institute Tasmania, University of Tasmania, Hobart, Tasmania, Australia; **A/Prof. Patricia Valery**, Menzies School of Health Research, Brisbane, Queensland, Australia; **A/Prof. Ingrid van der Mei**, Menzies Research Institute Tasmania, University of Tasmania, Hobart, Tasmania, Australia; **Dr. David Williams**, Hunter Health, Newcastle, New South Wales, Australia.

## Author Contributions

RR and VB analyzed the data and drafted the manuscript: JS, LB, AB, and RL critically reviewed the manuscript; RL and the Ausimmune Investigator Group designed and completed the Ausimmune Study. The content within the manuscript has not been published elsewhere and all authors are in agreement with the manuscript.

## Conflict of Interest Statement

The authors declare that the research was conducted in the absence of any commercial or financial relationships that could be construed as a potential conflict of interest.
