# Supplementary material for: Reported Changes in Dietary Behavior Following a First Clinical Diagnosis of Central Nervous System Demyelination
Source: Front Neurol. 2018 Mar 20;9:161. doi: 10.3389/fneur.2018.00161 (PMC5870150; doi:10.3389/fneur.2018.00161)
Supplement: Supplementary file 1 [file table_1.docx]

Supplementary Material

**Reported Changes in Dietary Behaviour Following a First Clinical Diagnosis of Central Nervous System Demyelination**

Rebecca D Russell^1^, Robyn M Lucas^2,3*^, Vanessa Brennan^1^, Jill Sherriff ^1^, Andrea Begley^1^, the Ausimmune Investigator Group, Lucinda J Black^1^

^1^ School of Public Health, Curtin University, Perth, WA, Australia

^2^ National Centre for Epidemiology and Population Health, Research School of Population Health, The Australian National University, Canberra, Australia

^3^ Centre for Ophthalmology and Visual Science, University of Western Australia, Perth, Western Australia

***Correspondence:**

Robyn M Lucas

Robyn.lucas@anu.edu.au

**Supplementary Table 1** Dietary changes reported by fewer than five participants in the one-year follow up of the Ausimmune Study

| **Description of dietary change** | **Number of participants reporting change** |
| --- | --- |
| Increased protein and/or meat | 4 |
| Meal replacement/weight loss diet | 4 |
| Overall healthier diet | 4 |
| Reduced energy intake, including smaller portions | 4 |
| Reduced or eliminated caffeine | 4 |
| Vitamin and/or mineral supplements | 4 |
| Increased dairy | 3 |
| Increased wholegrains | 3 |
| Reduced or eliminated bread | 3 |
| Increased chicken | 2 |
| Increased legumes | 2 |
| Increased nuts | 2 |
| Increased processed/convenience foods and/or reduced fresh food | 2 |
| Less regular meals or reduced snacking | 2 |
| Low Glycaemic Index diet^1^ | 2 |
| Addition of honey in tea and coffee | 1 |
| Addition of oils: omega-3, omega-6, flax seed and cod liver | 1 |
| Commenced drinking 2 glasses of wine per week | 1 |
| Commenced eating breakfast | 1 |
| Eliminated acidic foods | 1 |
| Eliminated lactose | 1 |
| Eliminated soy | 1 |
| Eliminated white bread | 1 |
| Genesis diet^2^ | 1 |
| Increased alcohol | 1 |
| Increased discretionary/sweet foods | 1 |
| Increased energy density of foods | 1 |
| Increased fibre | 1 |
| Increased muesli | 1 |
| Increased omega-3 intake | 1 |
| Increased prune juice | 1 |
| Increased tea and coffee | 1 |
| Increased vitamin D | 1 |
| Only consumed organic vegetables | 1 |
| Overall increased food intake | 1 |
| Reduced diet drinks | 1 |
| Reduced eggs | 1 |
| Reduced nuts | 1 |
| Reduced sodium intake | 1 |
| Soft food consumption only | 1 |
| Special MS diet (not further described) | 1 |
| Started skipping meals | 1 |
| Stopped skipping meals | 1 |
| Swank Diet^3^ | 1 |
| Vegan | 1 |
| NB. Participants could select more than one type of dietary change. | |

^1^ Low GI Diet [Internet]. Sydney (Australia): SP Health Co Pty Ltd; 2016 [cited 2017 Jun 22]. Available from: https://www.totalwellbeingdiet.com/the-diet/low-gi/

^2^ Weinblatt V. The Genesis Diet [Internet]. [place unknown]: Livestrong.com; 2015 [cited 2017 Jun 22]. Available from: http://www.livestrong.com/article/405719-the-genesis-diet/

^3^ Swank RL, Goodwin J. Review of MS patient survival on a Swank low saturated fat diet. *Nutrition* (2003) 19(2):161-2. doi: 10.1016/S0899-9007(02)00851-1
